# Demographic and clinical influences on emotion prosody recognition

**DOI:** 10.3389/fpsyg.2026.1900291

**Published:** 2026-08-25

**Authors:** Martin Simcik, Ross Andel, Jan Pavlik, Petr Marusic, Jana Amlerova, Jakub Hort

**Affiliations:** 1 Department of Neurology, Second Faculty of Medicine, Charles University, Motol and Homolka University Hospital (Motol University Hospital is a Full Member of ERN EpiCARE), Prague, Czechia; 2 Edson College of Nursing and Health Innovation, Arizona State University, Phoenix, AZ, United States; 3 AlzheimerChain Foundation, Prague, Czechia

**Keywords:** emotion recognition, health factors, individual differences, mobile app, prosody, social cognition

## Abstract

**Introduction:**

Emotion recognition from speech prosody is a complex cognitive ability that depends on the functional integrity of a distributed neuronal network and may be shaped by demographic, clinical and lifestyle factors. The Terrapino mobile application was developed as a tool to promote cognitive health, but its digital infrastructure and methodologically rigorous design also render it suitable for research purposes including a comprehensive battery to assess Alzheimer’s disease risk factors, as well as measures of perceived stress, metamemory, and prosody recognition.

**Methods:**

We analyzed data from 2,858 adult Terrapino users aged 18–103 years (median = 60 years) to identify correlates of emotional prosody recognition across a demographically, clinically and lifestyle-diverse population. Participants completed an emotion prosody recognition test comprising 25 audio recordings in which professional actors conveyed one of five basic emotions (fear, sadness, anger, disgust and happiness) solely through vocal intonation.

**Results:**

Consistent with previous research, age, sex, education, hearing problems, subjective cognitive complaints and the number of comorbidities were associated with performance, with several predictors significant in at least one age group. However, other hypothesized factors, such as alcohol consumption, depression, perceived stress, average hours of sleep, or living alone, did not show significant associations with performance in prosody recognition.

**Discussion:**

Together, these findings identify the main demographic and health factors associated with emotional prosody recognition across the adult lifespan and indicate that, in this large appbased cohort, performance is associated with age and health-related factors rather than with the lifestyle variables examined.

## Introduction

Prosody constitutes a fundamental component of human communication (Larrouy-Maestri et al., 2025). It encompasses the rhythm, tone, pitch, emphasis, and accent patterns of spoken language (Wagner and Watson, 2010), serving to convey information about others’ inner states and emotions in a non-verbal manner (Larrouy-Maestri et al., 2025; Banse and Scherer, 1996). Recognizing emotions is a vital aspect of social cognition (American Psychiatric Association, 2013), and therefore integral to social life as well (Frith and Frith, 2007). Impairments in prosody emotion recognition may contribute to unsuccessful psychosocial functioning that influence the overall quality of life (Hall et al., 2009).

Overall, women may exhibit better emotion recognition performance than men. A meta-analysis has provided conclusive evidence of a small yet significant female advantage in emotion recognition across both visual (facial emotion recognition) and auditory tasks (Thompson and Voyer, 2014). In addition to sex, age is another crucial factor influencing emotion recognition performance, as demonstrated by several studies (Ruffman et al., 2008; Olderbak et al., 2019; Abbruzzese et al., 2019), whereby younger people outperform older adults. More specifically, facial emotion recognition appears to reach its best performance peak between ages 15 and 30, after which it steadily declines (Olderbak et al., 2019). Age also appears to be a moderator of sex differences in emotion recognition. While females have been shown to outperform males since infancy (Thompson and Voyer, 2014), young adult females show the most pronounced advantage (Thompson and Voyer, 2014; Olderbak et al., 2019). This gap subsequently narrows between ages 46 and 60, before widening again in older age groups (Olderbak et al., 2019). Education also seems to play a role in emotion recognition. In the elderly, higher education has been associated with reduced atrophy in certain right-sided brain regions including the right supramarginal gyrus, which plays an important role in emotion recognition performance (Wada et al., 2021). Education and cognitive reserve may therefore be a factor that helps to preserve the structural integrity of specific brain regions, thereby supporting prolonged stability of emotion recognition during the process of aging.

Recent research has examined several other factors that influence emotional processing. For instance, older adults with hearing impairment demonstrate marked deficits in processing emotions from prosody, compared to their normally hearing peers (Christensen et al., 2019). Studies focusing on specific emotions reveal that depression impairs the recognition of most basic emotions, possibly with the exception of sadness (Dalili et al., 2014). Meanwhile, habitual alcohol consumption is associated with a tendency to misidentify facial expressions as hostile (angry, disgusted) (Freeman et al., 2018), and with difficulties recognizing sad and fearful expressions (Rajesh et al., 2021). The effects of social isolation on emotion recognition have also been investigated. However, findings remain inconclusive, with some studies reporting no association (Kanai et al., 2012), while others suggest either diminished emotion identification skills (Zysberg, 2012) or improved accuracy of recognizing anger in socially isolated individuals (Lodder et al., 2016). Notably, all of the aforementioned factors also represent established risk factors for dementia (Livingston et al., 2024).

While most previous studies have focused on each factor in isolation, our study simultaneously investigated a range of demographic factors (sex, age, education, physical activity, social isolation, and self-perceived stress) and clinical factors (hearing ability, alcohol consumption, depressive symptoms, sleep quality, and the number of comorbidities) to determine the most influential predictors of emotion prosody recognition (EPR). Additionally, instead of employing facial expressions, as is typical in prior research, an auditory test for prosody recognition was used in this study. We hypothesized that middle-aged adults would demonstrate the highest EPR performance, with the most pronounced sex differences emerging in the young adult group. Furthermore, we expected that factors traditionally associated with better cognitive aging, such as higher education, preserved hearing, absence of depressive symptoms, greater physical activity, fewer comorbidities, not living alone (Livingston et al., 2024) and lower levels of self-perceived stress (Franks et al., 2021), would positively influence EPR performance.

## Materials and methods

### Study design and participants

In this cross-sectional study, data were collected through the Terrapino mobile application (AlzheimerChain Foundation, 2022). Terrapino is a comprehensive and interactive platform designed for promotion of healthy lifestyle and for the acquisition of clinical and demographic information, with a primary focus on data relevant to prevention of Alzheimer’s disease. The application was developed based on theoretical frameworks such as the Technology Acceptance Model (Venkatesh et al., 2003) and Human-Centered Design (An et al., 2023; Nimmanterdwong et al., 2022) to ensure it is both user-friendly and methodologically robust for research purposes. Users were prompted to complete an anonymous questionnaire that captured demographic characteristics, clinical history, and lifestyle factors. In addition, the Terrapino application includes cognitive training tasks, puzzles, accessible health related tips, rewards and standardized assessments, including the Emotion Prosody Recognition (EPR) test, which constituted the core measure in the present study. Participation was voluntary and the sample was therefore not recruited using random sampling procedures. A total of 2,904 adult users completed both the questionnaire and the EPR test during the data collection period, which spanned from December 24, 2022, to December 31, 2024. Participants who self-reported a diagnosis of Alzheimer’s disease or other related dementia (*n* = 46) were excluded from the analyses. The final sample therefore comprised 2,858 participants. The test was made available in both Czech and English, but the vast majority completed the Czech version, with only 134 participants opting for the English version. Although understanding the content of the presented sentences was not necessary, as the task intentionally minimizes semantic information, all participants completed the task in their respective native language.

### Measures

#### Questionnaire

Demographic factors included age, sex, education, and living arrangement (alone/not alone). Clinical history information included the self-perceived stress scale adapted from Cohen et al. (1983), self-reported depressive symptoms, hearing problems, subjective cognitive complaints, as well as total number of comorbidities. With regard to lifestyle factors, participants provided information on dietary habits, physical activity, alcohol use, and hours of sleep per night. The complete list of queried comorbidities as well as the complete list of questions used to assess subjective cognitive complaints is provided in Appendix.

#### Emotion prosody recognition

To assess emotion recognition abilities, we employed the EPR test. The Czech paradigm was developed according to the methodology described by Ariatti et al. (2008). The original stimulus set consisted of 200 audio recordings that were normatively evaluated by 88 healthy volunteers. Just 25 recordings that most reliably conveyed the emotional categories were selected in the final version of EPR test. The same EPR paradigm has previously been used in clinical studies including healthy control groups and patients with Alzheimer’s disease or amnestic mild cognitive impairment (Amlerova et al., 2022) as well as patients with temporal lobe epilepsy (Amlerova et al., 2026) thereby providing reference performance data for the task. The EPR test used in the present study comprises 25 audio recordings, each approximately 3 s in duration. The recordings were produced by professional Czech actors (two males and two females, all native speakers), expressing one of five basic emotions (sadness, fear, happiness, disgust, or anger) exclusively through vocal intonation. Surprise was not included as a target emotion in the EPR paradigm to maintain consistency with the modified Face Emotion Recognition Test used in previous work, in which surprise had been removed because it was frequently misidentified as fear (Rapcsak et al., 2000). The spoken content of each recording was emotionally neutral, using one of two standardized Czech sentences: “The table has four legs” and “A barking dog does not bite.” These sentences were selected to ensure that emotional cues were derived solely from prosody rather than semantic content. Each of the five target emotions was represented by five recordings, although this information was not disclosed to the participants. Responses were scored dichotomously as either correct or incorrect, yielding a total possible score ranging from 0 to 25 points. There was no time limit to complete the task. Correct answers were not given to participants.

### Analyses

Participants were first stratified into four age groups: (1) 18–39 years, (2) 40–59 years, (3) 60–74 years, and (4) 75 + years. This stratification was implemented to maintain adequate sample sizes across strata and to enable a more granular assessment of age-related associations, particularly in older adulthood, extending prior work in which populations were either dichotomized only into two groups (i.e., the young and the old) (Olderbak et al., 2019), or capped the categorization at 60 + years of age, without further differentiation for older age ranges (Abbruzzese et al., 2019). We then fitted the same prespecified multiple linear regression model separately within each age stratum to examine associations between the outcome (EPR score) and a hypothesis-driven set of predictors. Candidate predictors were selected *a priori* based on previous evidence linking them to emotion recognition performance and on their established relevance as risk factors for cognitive decline: sex, educational attainment, alcohol consumption, living arrangement (living alone vs. not alone), depressive symptoms, physical activity, perceived stress, subjective cognitive complaints, hearing problems, dietary habits, total number of comorbidities, and average sleep duration.

Prior to conducting the analysis, we assessed key statistical assumptions: normal distribution of residuals (Q-Q plot), linearity (residuals-fitted plot), homoscedasticity (Breusch-Pagan test). All participants in the analytic sample had complete data for the variables included in the regression models, therefore no participants were excluded because of missing data and no imputation was performed. Multicollinearity was negligible, with maximum VIF values ranging from 1.34 to 1.75 across the four age-stratified models. Breusch-Pagan tests indicated heteroscedasticity in the 18–39 and 75 + models. For comparison, we therefore calculated robust standard errors using the HC3 method (Hayes and Cai, 2007). In the final reporting, regression coefficients are presented as unstandardized estimates, whereas standard errors, 95% confidence intervals, and *p*-values are based on HC3 robust standard errors. No formal correction for multiple testing was applied across regression coefficients or age-stratified models. Because the analyses were hypothesis-driven, used a single outcome variable, and included the same *a priori* selected predictors within each multivariable model, a global adjustment was considered overly conservative and potentially likely to increase the risk of Type II error.

### Transparency and openness

The study used anonymized data collected without intervention. The sample size was determined based on the available dataset. We report how we determined our sample size, all data exclusions and manipulations, and all measures in the study. All statistical analyses were conducted using R version 4.4.3 (R Core Team, 2024). The source data sheet and the analysis code are available at https://doi.org/10.5281/zenodo.19036606. This study’s design and its analysis were not pre-registered.

## Results

### Demographics

Descriptive statistics for each age group are summarized in Table 1. The final analytic sample included 2,858 participants, ranging in age from 18 to 103 years. The majority of participants were female (77.6%), and the male to female ratio was proportionally distributed across all age groups. The youngest (*n* = 275) and oldest (*n* = 295) age groups comprised the smallest cohorts, whereas the largest were those aged 40–59 years (*n* = 1,153) and 60–74 years (*n* = 1,135).

**Table 1:** Descriptive characteristics of the study sample by age group and sex.

Age group	All	18–39 years		40–59 years		60–74 years		75 + years	
Sex		Females	Males	Females	Males	Females	Males	Females	Males
*n*	2,858	201	74	888	265	902	233	226	69
Proportion, %	77.6 (Females)	73.1	26.9	77	23	79.5	20.5	76.6	23.4
Age (years), mean (SD)	58.2 (13.9)	30.6 (5.6)	30.7 (5.9)	51.1 (5.3)	49.9 (5.2)	67.2 (4.1)	66.8 (4.2)	78.6 (3.7)	78.6 (4.3)
Education, %									
Elementary or lower	8.6	5.5	16.2	7.3	14.7	7.5	12.9	7.1	5.8
Trade school	44.3	35.3	35.1	45.4	40.8	49.9	27.9	53.5	33.3
High school	44	57.2	47.3	44.6	40.4	40.1	53.2	35.8	53.6
College or higher	3.1	2	1.4	2.7	4.2	2.4	6	3.5	7.2
Alone, %	29.2	33.8	52.7	23.6	15.5	35.4	12.4	53.5	11.6
Depression, %	14.3	10.9	14.9	15.9	12.5	16	7.3	15.9	8.7
Self-perceived stress scale, mean (SD)	26.3 (6.1)	22.2 (6.6)	23.9 (7.3)	25.5 (6.3)	26.3 (5.9)	27.5 (5.5)	28.2 (5.8)	26.5 (5.4)	27.9 (5)
Subjective cognitive complaints, mean (SD)	1.8 (1.6)	1.5 (1.5)	2.1 (1.9)	1.8 (1.6)	1.7 (1.7)	1.7 (1.5)	1.8 (1.6)	2.1 (1.7)	2.5 (2)
Hearing problems, %	21.6	11.3	9.4	16.6	18.9	23.1	27.1	42.2	55.1
Comorbidities, mean (SD)	1.1 (1.1)	0.4 (0.7)	0.4 (0.6)	0.8 (0.9)	0.8 (1)	1.3 (1.1)	1.5 (1.1)	1.8 (1.2)	1.8 (1.2)
Drinks alcohol, %	68.1	71.1	58.1	68.9	61.9	70.2	64.4	68.6	66.7
Dietary habits (score), mean (SD)	6.4 (1.4)	6.2 (1.5)	5.6 (1.7)	6.5 (1.4)	5.9 (1.5)	6.6 (1.3)	6.1 (1.5)	6.5 (1.3)	5.7 (1.2)
Physical activity (score), mean (SD)	2.3 (1.3)	2.3 (1.3)	2.3 (1.6)	2.3 (1.3)	2.2 (1.4)	2.4 (1.3)	2.2 (1.3)	2.2 (1.3)	2.1 (1.4)
Sleep (hours), mean (SD)	7.1 (1.1)	7.2 (1.1)	7.2 (1.3)	7 (1.0)	6.9 (1.2)	7.1 (1.1)	7.2 (1.1)	7.2 (1.2)	7.4 (1.3)

SD, standard deviation.

### Multivariate linear regression analysis

**Figure 1:**
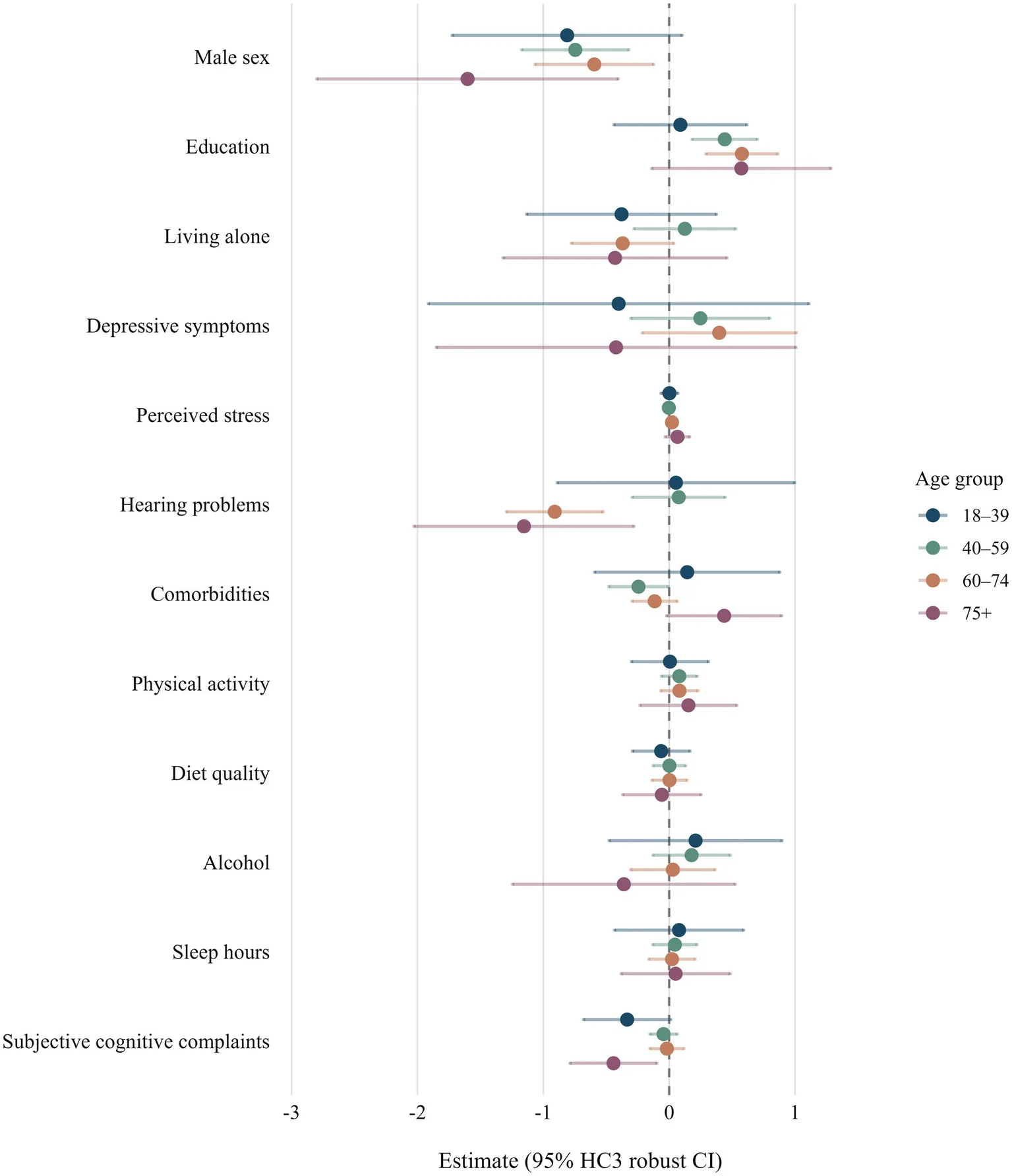
Regression estimates and 95% confidence intervals for predictors of emotion prosody recognition across age groups.

The results are summarized in Table 2 and visualized in Figure 1. Several variables were significantly associated with EPR performance in at least one age group, including sex, education, hearing problems, number of comorbidities, and subjective cognitive complaints. In contrast, alcohol consumption, living alone, depressive symptoms, hours of sleep, dietary habits, physical activity, and perceived stress did not demonstrate a significant association with EPR performance in any age group. In the youngest age group (18–39 years), the assumption of homoscedasticity was violated. When HC3 robust standard errors were applied, none of the examined predictors remained significantly associated with EPR performance.

**Table 2:** Multivariable linear regression models predicting emotion prosody recognition performance by age group.

Predictor	18–39				40–59				60–74				75+			
	Estimate	CI low	CI high	*p-*value	Estimate	CI low	CI high	*p-*value	Estimate	CI low	CI high	*p-*value	Estimate	CI low	CI high	*p-*value
(Intercept)	20.3	17.1	23.5	<0.001	18.6	16.9	20.3	<0.001	16.9	15.0	18.7	<0.001	13.9	9.6	18.3	<0.001
Male sex	−0.8	−1.7	0.1	0.081	−0.7	−1.2	−0.3	<0.001	−0.6	−1.1	−0.1	0.013	−1.6	−2.8	−0.4	0.009
Education	0.1	−0.4	0.6	0.740	0.4	0.2	0.7	<0.001	0.6	0.3	0.9	<0.001	0.6	−0.1	1.3	0.114
Living alone	−0.4	−1.1	0.4	0.323	0.1	−0.3	0.5	0.545	−0.4	−0.8	0.0	0.075	−0.4	−1.3	0.5	0.341
Depressive symptoms	−0.4	−1.9	1.1	0.601	0.2	−0.3	0.8	0.377	0.4	−0.2	1.0	0.202	−0.4	−1.8	1.0	0.561
Self-perceived stress	0.0	−0.1	0.1	0.947	−0.0	−0.0	0.0	0.865	0.0	−0.0	0.1	0.231	0.1	−0.0	0.2	0.180
Hearing problems	0.1	−0.9	1.0	0.910	0.1	−0.3	0.4	0.686	−0.9	−1.3	−0.5	<0.001	−1.2	−2.0	−0.3	0.010
Comorbidities	0.1	−0.6	0.9	0.701	−0.2	−0.5	−0.0	0.043	−0.1	−0.3	0.1	0.205	0.4	−0.0	0.9	0.060
Physical activity	0.0	−0.3	0.3	0.968	0.1	−0.1	0.2	0.264	0.1	−0.1	0.2	0.276	0.2	−0.2	0.5	0.433
Dietary habits	−0.1	−0.3	0.2	0.582	0.0	−0.1	0.1	0.976	0.0	−0.1	0.1	0.968	−0.1	−0.4	0.3	0.713
Alcohol	0.2	−0.5	0.9	0.547	0.2	−0.1	0.5	0.254	0.0	−0.3	0.4	0.864	−0.4	−1.2	0.5	0.423
Hours slept	0.1	−0.4	0.6	0.763	0.0	−0.1	0.2	0.609	0.0	−0.2	0.2	0.810	0.1	−0.4	0.5	0.815
Subjective cognitive complaints	−0.3	−0.7	0.0	0.058	−0.0	−0.2	0.1	0.412	−0.0	−0.2	0.1	0.791	−0.4	−0.8	−0.1	0.012

CI, confidence interval.

**Figure 2:**
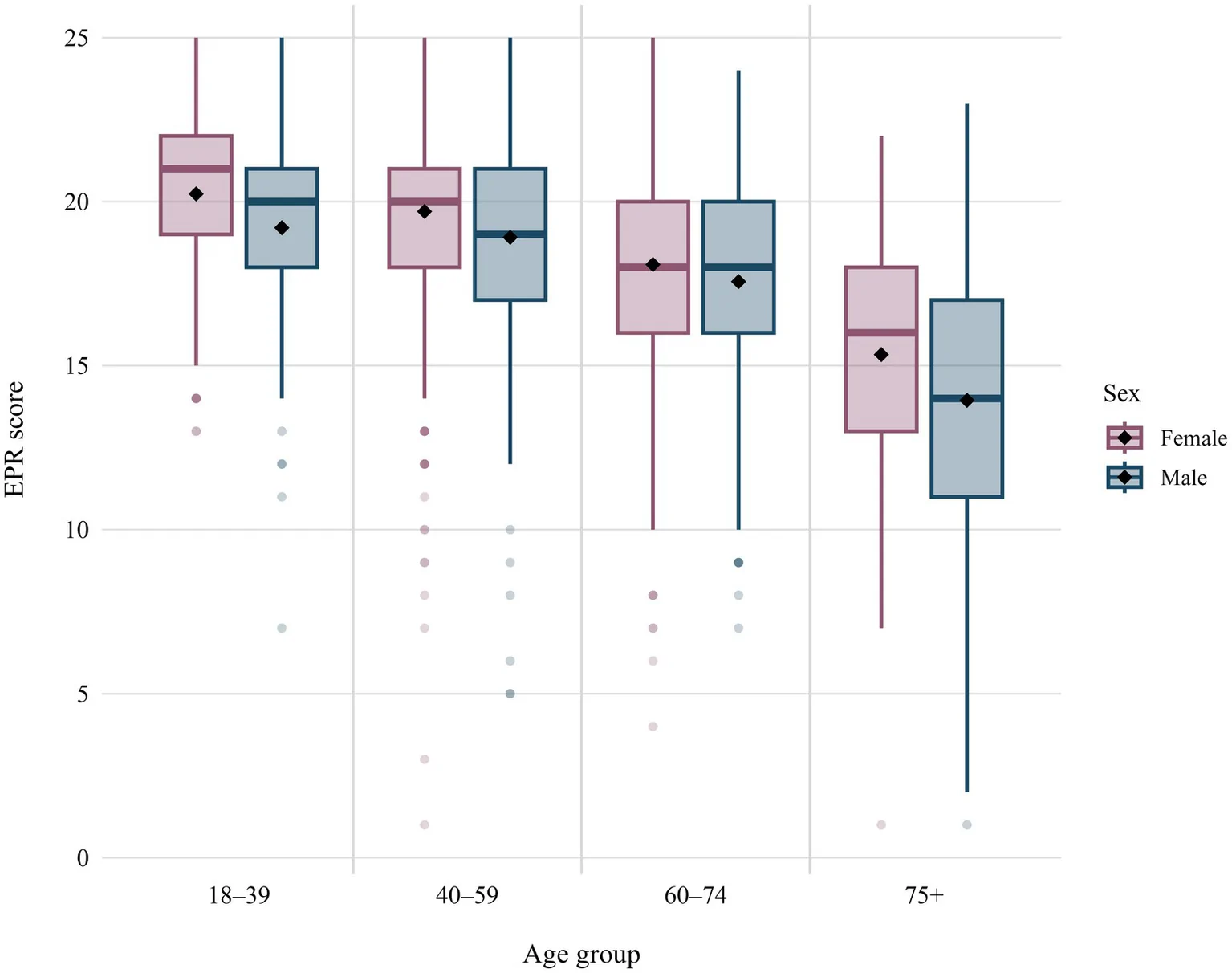
Emotion prosody recognition scores by age group and sex.

EPR performance exhibited a progressive decline across age groups (Figure 2). The highest mean score was observed in the youngest cohort (18–39 years; estimate = 20.3, *p* < 0.001) followed by approximately a 2-point decrement in the older age groups (40–59: estimate = 18.6, *p* < 0.001; 60–74: estimate = 16.9, *p* < 0.001). The most substantial decline was noted in the oldest group (75 + years; estimate = 13.9, *p* < 0.001).

A female advantage in EPR performance was observed in the three older age groups, but not in the youngest group, where the association between male sex and lower EPR performance did not reach statistical significance (estimate = −0.8, *p* = 0.081). In the 40–59 and 60–74 groups, males scored significantly lower than females, with effects of comparable magnitude (40–59 years: estimate = −0.7, *p* < 0.001; 60–74 years: estimate = −0.6, *p* = 0.013). While sex-related differences appeared to decrease from midlife to older adulthood, the oldest age group (75 + years) showed a notable reversal of this trend with females showing a significantly greater advantage (estimate = −1.6, *p* = 0.009).

Educational attainment was positively associated with EPR scores in the 40–59 and 60–74 groups. In the 18–39 group, education showed a small and non-significant association (estimate = 0.1, *p* = 0.740), whereas in the oldest group, education showed a larger effect but still did not reach significance (estimate = 0.6, *p* = 0.114). In contrast, for individuals aged 40–59, each incremental education level corresponded to a significant increase in EPR scores (estimate = 0.4, *p* < 0.001), with the strongest effect observed in the 60–74 group (estimate = 0.6, *p* < 0.001).

Hearing impairment was significantly associated with lower EPR performance, but only among the two oldest age groups. Among younger individuals, the effect was non-significant (18–39: estimate = 0.1, *p* = 0.910; 40–59: estimate = 0.1, *p* = 0.686). Conversely, hearing impairment predicted significantly lower EPR performance in the older groups (60–74: estimate = −0.9, *p* < 0.001; 75+: estimate = −1.2, *p* = 0.010).

The relationship between the number of comorbidities and EPR performance varied across age groups. In the youngest group, comorbidity was unrelated to EPR (estimate = 0.1, *p* = 0.701), whereas in the 40–59 group, each additional health condition predicted a drop in performance (estimate = −0.2, *p* = 0.043). No significant effect was found in the 60–74 cohort (estimate = −0.1, *p* = 0.205). In the oldest group (75+), the association was positive in direction but did not reach statistical significance after applying HC3 robust standard errors (estimate = 0.4, *p* = 0.060).

Subjective cognitive complaints were not significantly associated with EPR performance in participants younger than 75 years. In the youngest group (18–39 years), the association was negative in direction but did not reach statistical significance after applying HC3 robust standard errors (estimate = −0.3, *p* = 0.058). No significant associations were observed in the 40–59 group (estimate = −0.0, *p* = 0.412) nor the 60–74 group (estimate = −0.0, *p* = 0.791). In contrast, among participants aged 75 years and older, subjective cognitive complaints were significantly associated with lower EPR performance (estimate = −0.4, *p* = 0.012).

## Discussion

This study examined factors associated with EPR across the adult lifespan using data collected from a freely available, app-based prosody emotion recognition test. Our findings replicate and extend previous evidence by demonstrating that EPR performance declines with age (Yıldırım et al., 2024), with decrements evident as early as midlife (40–59 years) and most pronounced in the oldest age group (75 + years). The steepest drop in EPR performance was observed between the 60–74 and 75 + cohorts, aligning with known age-related degradation of neural networks responsible for emotional processing, particularly in frontotemporal regions (Ruffman et al., 2008; Raz et al., 2005). Consistent with prior meta-analyses (Thompson and Voyer, 2014; Lambrecht et al., 2014), we observed a robust and consistent female advantage in EPR performance, although this association did not reach significance in the youngest age group after robust correction. This sex effect was particularly marked in the oldest participants (75+), possibly reflecting sex differences in brain aging and cognition, with women exhibiting greater gray matter thickness (Luders et al., 2006) and less pronounced cortical thinning (Pacheco et al., 2015), especially in frontal and temporal regions (Cowell et al., 1994). Interestingly, the magnitude of the sex gap narrowed with age before reversing and subsequently again widening in late life, a pattern also noted in studies of facial emotion recognition (Olderbak et al., 2019).

Educational attainment emerged as a positive predictor of EPR in the middle aged (40–59) and older adult groups (60–74). This finding supports the notion that education may serve as a cognitive reserve factor (Stern, 2012), potentially mitigating age-related decline in socioemotional abilities through the preservation of function in key neural circuits, including the right supramarginal gyrus (Wada et al., 2021). Although the education estimate in the 75 + group was comparable in magnitude to that observed in the 60–74 group, it did not reach statistical significance, likely reflecting reduced statistical power and greater interindividual variability in very late life, where sensory, cognitive, and health-related factors may obscure education-related reserve effects.

Hearing problems were significantly associated with lower EPR performance in the two oldest age groups, aligning with prior research linking sensory deficits to communication difficulties, misinterpretation of social cues (Heine and Browning, 2004), and increased risk of cognitive decline (Livingston et al., 2024). Large-scale surveys further suggest that hearing loss impacts social functioning more profoundly than visual impairment (Pinto et al., 2014). Together with our findings, this underscores the critical role of intact auditory processing in recognizing emotional cues in speech and raises concerns about the often underappreciated socioemotional consequences of hearing impairment in older adults. Furthermore, because sensory deficits become more prevalent with age (Völter et al., 2021), older adults increasingly rely on multisensory cues (Hunter et al., 2010) - while emotion recognition from a single modality (e.g., faces or voices alone) declines with age, performance can match that of younger adults when congruent multisensory information is available. As our study used only a single-modality task (EPR), this aspect should be considered when interpreting the results.

Subjective cognitive complaints were negatively associated with EPR only in the oldest (75+) age group. In the youngest group (18–39 years), the association was negative in direction but did not remain statistically significant after applying HC3 robust standard errors and should therefore be interpreted with caution. Prior research suggests that subjective cognitive complaints in younger adults are often driven by psychosocial and affective factors such as stress, negative mood, or poor sleep (Jenkins et al., 2025; Lee et al., 2020), without necessarily being accompanied by objective cognitive impairment (Csábi et al., 2025). Factors such as negative mood are also known to interfere with recognizing emotions (Dalili et al., 2014; Scibelli et al., 2016) and may therefore contribute to reduced prosody emotion recognition. However, because we did not analyze the interaction between subjective cognitive complaints and depressive symptoms in our study, this interpretation should be considered tentative. In contrast, subjective cognitive complaints in older adults are frequently interpreted as an early risk of future objective cognitive decline, MCI, and dementia (Wang et al., 2021). This framework provides a plausible explanation for the negative association between EPR scores and the extent of subjective cognitive complaints, which we observed in the 75 + group. The finding is consistent with the view that declining emotion recognition may accompany broader early changes during preclinical stages of neurodegenerative disease.

The number of comorbidities was negatively associated with EPR uniquely in the 40–59 group. In the oldest group (75 + years), the association was positive in direction but did not reach significance. Given the substantial evidence linking a higher burden of chronic disease to cognitive decline (Wei et al., 2020; Wang et al., 2025; Zhou et al., 2025), it is notable that this expected negative association was observed only in the 40–59 group, which is consistent with the view that accumulated health burden may adversely affect cognitive and socioemotional functioning. However, the absence of significant association in the two older groups suggests that this relationship may weaken in later life or may be influenced by other age-related factors. One possible explanation is the protected survivor model, whereby individuals who survive to a very advanced age despite a high burden of risk factors may represent a more resilient subgroup (Silverman and Schmeidler, 2018). In such populations, the typical relationship between risk factors and adverse outcomes may weaken, not because risk factors become beneficial, but because high-risk survivors may possess unmeasured biological, cognitive, or psychosocial protective characteristics. These individuals are therefore an example of resilient successful cognitive aging, which is defined as preserved cognitive function despite substantial risk exposure (Silverman and Schmeidler, 2018). However, because the positive association in the 75 + group was not statistically significant in the robust analysis, this interpretation remains speculative and should be examined in future more robust studies.

Contrary to some expectations, lifestyle-related variables such as physical activity, diet, sleep duration, alcohol use, depressive symptoms, living arrangement, and perceived stress were not significantly associated with EPR in any age group. While previous studies have linked some of these factors to cognitive aging (Livingston et al., 2024; Franks et al., 2021), their influence on socioemotional processing, particularly prosody recognition, may be more subtle or moderated by other variables not captured in this study design (e.g., quality of social interaction, affective empathy, or neuroinflammatory markers).

Several limitations should be acknowledged. First, the cross-sectional design does not allow us to determine the direction of the observed associations. Longitudinal data are needed to clarify whether changes in EPR precede, coincide with, or follow broader cognitive decline. Second, all measures (aside from the EPR test) relied on self-report, which may be subject to recall bias or social desirability effects, particularly in sensitive domains such as depressive symptoms, alcohol consumption, and cognitive complaints. In addition, participants were not recruited through random sampling procedures but voluntarily used a mobile application focused on cognitive health. This may have enriched the sample for individuals who are more health-conscious, digitally literate, cognitively engaged, and motivated to complete cognitive tasks. Conversely, individuals with lower digital literacy, lower socioeconomic status, or reduced access to smartphone technology may have been underrepresented. This may be particularly relevant for the oldest age group, in which the use of smartphones may reflect a selected subgroup with higher functional independence or cognitive reserve. The sample was also predominantly female, which may further limit generalizability. Therefore, the present findings should not be interpreted as causes for changes in EPR performance, but rather as associations observed within a large, self-selected app-based cohort.

An additional limitation is that several potentially relevant confounding variables were not assessed, including socioeconomic status, medication use, neurological disorders other than dementia, and objective hearing status. The current regression models explained only a limited proportion of variance, with R^2^ values ranging from approximately 4–14%. This suggests that the observed associations capture only part of the complex ability of emotion perception and recognition, and that other unmeasured factors may also have contributed to the findings. Future studies should include more comprehensive clinical and socioeconomic assessments to better account for these potential confounds.

Overall, our findings highlight the complexity of emotion recognition across the adult lifespan and suggest that EPR is associated with both stable demographic and clinical characteristics (sex, education, hearing ability) and dynamic age-related cognitive processes (e.g., subjective decline). The absence of significant associations with modifiable lifestyle factors warrants further investigation using longitudinal and experimental designs.

## Data Availability

The datasets presented in this study can be found in online repositories. The names of the repository/repositories and accession number(s) can be found in the article/supplementary material.

## Appendix

*The complete list of questions used to assess subjective cognitive complaints*

1. “In the past six months, have you felt like you've been having trouble remembering things, or have your close friends told you, ‘I already told you that’?”
2. “Have you forgotten an important meeting in the last six months?”
3. “Have you lost your belongings more often or for longer periods of time than usual in the last six months?”
4. “In the last six months, have you had trouble finding your way around or not recognizing a place you have already been to?”
5. “Have you completely forgotten an event in the last six months, even though your loved ones have told you about it or you have seen it in photographs?”
6. “In the last six months, have you had trouble finding the right words in conversation, used substitute words or said ‘it’ more often?”
7. “In the last six months, have you cut back on any personal or work activities or asked others for help because you were worried you would make a mistake?”
8. “In the past six months, have you noticed a change in your personality—becoming more withdrawn, reducing contact with others, losing interest in things, or showing less initiative?”

*The complete list of queried comorbidities*

- Alzheimer’s disease or another type of dementia
- stroke or other cerebrovascular insult
- concussion or severe head injury
- depression
- hearing loss
- high cholesterol
- oncological disease
- hypertension
- hypotension
- diabetes
- ischemic heart disease
- myocardial infarction
- COVID-19 during the previous year
- antibiotic use during the previous year

## References

[ref1] Abbruzzese L. Magnani N. Robertson I. H. Mancuso M. (2019). Age and gender differences in emotion recognition. Front. Psychol. 10:10(OCT). doi: 10.3389/FPSYG.2019.02371, 31708832 PMC6819430

[ref2] AlzheimerChain Foundation Terrapino (2022). Available online at: www.terrapino.com (Accessed March 28, 2025).

[ref3] American Psychiatric Association (2013). “Diagnostic and statistical manual of mental disorders,” in Diagn Stat Man Ment Disord, (Arlington, VA: American Psychiatric Association).

[ref4] Amlerova J. Laczó J. Nedelska Z. Laczó M. Vyhnálek M. Zhang B. . (2022). Emotional prosody recognition is impaired in Alzheimer’s disease. Alzheimer's Res. Ther. 14:50. 35382868 10.1186/s13195-022-00989-7 PMC8985328

[ref5] Amlerova J. Pytelova V. Angelucci F. Andel R. Hort J. Javurkova A. . (2026). Emotion recognition deficits in temporal lobe epilepsy are non-lateralized and independent of surgery. Epilepsy Behav. 176, 110906. doi: 10.1016/j.yebeh.2026.110906, 41576838

[ref6] An Q. Kelley M. M. Hanners A. Yen P. Y. (2023). Sustainable development for Mobile health apps using the human-centered design process. JMIR Form Res. 7:e45694. doi: 10.2196/45694, 37624639 PMC10492175

[ref7] Ariatti A. Benuzzi F. Nichelli P. (2008). Recognition of emotions from visual and prosodic cues in Parkinson’s disease. Neurol. Sci. 29, 219–227. doi: 10.1007/s10072-008-0971-9, 18810595

[ref8] Banse R. Scherer K. R. (1996). Acoustic profiles in vocal emotion expression. J. Pers. Soc. Psychol. 70, 614–636. doi: 10.1037/0022-3514.70.3.614, 8851745

[ref9] Christensen J. A. Sis J. Kulkarni A. M. Chatterjee M. (2019). Effects of age and hearing loss on the recognition of emotions in speech. Ear Hear. 40, 1069–1069. doi: 10.1097/AUD.0000000000000694, 30614835 PMC6606405

[ref10] Cohen S. Kamarck T. Mermelstein R. (1983). A global measure of perceived stress. J. Health Soc. Behav. 24, 385–396. doi: 10.2307/2136404 6668417

[ref11] Cowell P. E. Turetsky B. I. Gur R. C. Grossman R. I. Shtasel D. L. Gur R. E. (1994). Sex differences in aging of the human frontal and temporal lobes. J. Neurosci. 14, 4748–4755. doi: 10.1523/JNEUROSCI.14-08-04748.1994, 8046448 PMC6577197

[ref12] Csábi E. Kovács F. M. Volosin M. (2025). Predictors of subjective memory functioning in young adults. BMC Psychiatry 25:155. doi: 10.1186/s12888-025-06568-y 39972297 PMC11841223

[ref13] Dalili M. N. Penton-Voak I. S. Harmer C. J. Munafò M. R. (2014). Meta-analysis of emotion recognition deficits in major depressive disorder. Psychol. Med. 45, 1135–1135. doi: 10.1017/S0033291714002591, 25395075 PMC4712476

[ref14] Franks K. H. Bransby L. Saling M. M. Pase M. P. (2021). Association of Stress with risk of dementia and mild cognitive impairment: a systematic review and Meta-analysis. J Alzheimers Dis JAD. 82, 1573–1590. doi: 10.3233/JAD-210094, 34366334

[ref15] Freeman C. R. Wiers C. E. Sloan M. E. Zehra A. Ramirez V. Wang G. J. . (2018). Emotion recognition biases in alcohol use disorder. Alcohol. Clin. Exp. Res. 42, 1541–1547. doi: 10.1111/acer.13802, 29975424

[ref16] Frith C. D. Frith U. (2007). Social cognition in humans. Curr. Biol. 17, R724–R732. doi: 10.1016/J.CUB.2007.05.068 17714666

[ref17] Hall J. A. Andrzejewski S. A. Yopchick J. E. (2009). Psychosocial correlates of interpersonal sensitivity: a meta-analysis. J. Nonverbal Behav. 33, 149–180. doi: 10.1007/s10919-009-0070-5

[ref18] Hayes A. F. Cai L. (2007). Using heteroskedasticity-consistent standard error estimators in OLS regression: An introduction and software implementation. Behav. Res. Methods 39, 709–722. doi: 10.3758/BF03192961, 18183883

[ref19] Heine C. Browning C. J. (2004). The communication and psychosocial perceptions of older adults with sensory loss: a qualitative study. Ageing Soc. 24, 113–130. doi: 10.1017/S0144686X03001491

[ref20] Hunter E. M. Phillips L. H. MacPherson S. E. (2010). Effects of age on cross-modal emotion perception. Psychol. Aging 25, 779–787. doi: 10.1037/a0020528 21186914

[ref21] Jenkins A. Tree J. Tales A. (2025). Distinct profile differences in subjective cognitive decline in the general public are associated with metacognition, negative affective symptoms, neuroticism, stress, and poor quality of life. J. Alzheimer's Dis 80, 1231–1242. doi: 10.3233/JAD-200882, 33646150 PMC8150446

[ref22] Kanai R. Bahrami B. Duchaine B. Janik A. Banissy M. J. Rees G. (2012). Brain structure links loneliness to social perception. Curr. Biol. 22, 1975–1979. doi: 10.1016/J.CUB.2012.08.045, 23041193 PMC3510434

[ref23] Lambrecht L. Kreifelts B. Wildgruber D. (2014). Gender differences in emotion recognition: impact of sensory modality and emotional category. Cogn Emot. 28, 452–469. doi: 10.1080/02699931.2013.837378, 24151963

[ref24] Larrouy-Maestri P. Poeppel D. Pell M. D. (2025). The sound of emotional prosody: nearly 3 decades of research and future directions. Perspect. Psychol. Sci. 20, 623–638. doi: 10.1177/17456916231217722 38232303 PMC12231869

[ref25] Lee J. E. Ju Y. J. Park E. C. Lee S. Y. (2020). Effect of poor sleep quality on subjective cognitive decline (SCD) or SCD-related functional difficulties: results from 220,000 nationwide general populations without dementia. J. Affect. Disord. 260, 32–37. doi: 10.1016/j.jad.2019.08.082, 31493636

[ref26] Livingston G. Huntley J. Liu K. Y. Costafreda S. G. Selbæk G. Alladi S. . (2024). Dementia prevention, intervention, and care: 2024 report of the lancet standing commission. Lancet 404, 572–628. 39096926 10.1016/S0140-6736(24)01296-0

[ref27] Lodder G. M. A. Scholte R. H. J. Goossens L. Engels R. C. M. E. Verhagen M. (2016). Loneliness and the social monitoring system: emotion recognition and eye gaze in a real-life conversation. Br. J. Psychol. 107, 135–153. doi: 10.1111/BJOP.12131, 25854912

[ref28] Luders E. Narr K. L. Thompson P. M. Rex D. E. Woods R. P. Deluca H. . (2006). Gender effects on cortical thickness and the influence of scaling. Hum. Brain Mapp. 27, 314–324. doi: 10.1002/hbm.20187, 16124013 PMC6871390

[ref29] Nimmanterdwong Z. Boonviriya S. Tangkijvanich P. (2022). Human-centered Design of Mobile Health Apps for older adults: systematic review and narrative synthesis. JMIR Mhealth Uhealth 10:e29512. doi: 10.2196/29512, 35029535 PMC8800094

[ref30] Olderbak S. Wilhelm O. Hildebrandt A. Quoidbach J. (2019). Sex differences in facial emotion perception ability across the lifespan. Cogn Emot. 33, 579–588. doi: 10.1080/02699931.2018.1454403, 29564958

[ref31] Pacheco J. Goh J. O. Kraut M. A. Ferrucci L. Resnick S. M. (2015). Greater cortical thinning in normal older adults predicts later cognitive impairment. Neurobiol. Aging 36, 903–908. doi: 10.1016/j.neurobiolaging.2014.08.031, 25311277 PMC4315712

[ref32] Pinto J. M. Kern D. W. Wroblewski K. E. Chen R. C. Schumm L. P. McClintock M. K. (2014). Sensory function: insights from wave 2 of the National Social Life, health, and aging project. J. Gerontol. Ser. B. 69, S144–S153. doi: 10.1093/geronb/gbu102 PMC4303092 25360015

[ref33] R Core Team (2024). R: A Language and Environment for Statistical Computing. Vienna, Austria: R Foundation for Statistical Computing.

[ref34] Rajesh C. L. Endreddy A. R. Shaik S. Venu Gopala Raju S. V. (2021). A comparative study of facial emotion recognition pattern and its determinants in patients of alcohol dependence syndrome versus matching controls. Ann. Indian Psychiatry 5, 61–66. doi: 10.4103/AIP.AIP_136_20

[ref35] Rapcsak S. Z. Galper S. R. Comer J. F. Reminger S. L. Nielsen L. Kaszniak A. W. . (2000). Fear recognition deficits after focal brain damage: a cautionary note. Neurology 54, 575–581. doi: 10.1212/WNL.54.3.575 10680785

[ref36] Raz N. Lindenberger U. Rodrigue K. M. Kennedy K. M. Head D. Williamson A. . (2005). Regional brain changes in aging healthy adults: general trends, individual differences and modifiers. Cereb. Cortex 15, 1676–1689. doi: 10.1093/cercor/bhi044, 15703252

[ref37] Ruffman T. Henry J. D. Livingstone V. Phillips L. H. (2008). A meta-analytic review of emotion recognition and aging: implications for neuropsychological models of aging. Neurosci. Biobehav. Rev. 32, 863–881. doi: 10.1016/J.NEUBIOREV.2008.01.001, 18276008

[ref38] Scibelli F. Troncone A. Likforman-Sulem L. Vinciarelli A. Esposito A. (2016). How major depressive disorder affects the ability to decode multimodal dynamic emotional stimuli. Front. ICT 3:16. doi: 10.3389/fict.2016.00016

[ref39] Silverman J. M. Schmeidler J. (2018). The protected survivor model: using resistant successful cognitive aging to identify protection in the very old. Med. Hypotheses 110, 9–14. doi: 10.1016/j.mehy.2017.10.022, 29317078 PMC5927359

[ref40] Stern Y. (2012). Cognitive reserve in ageing and Alzheimer’s disease. Lancet Neurol. 11, 1006–1012. doi: 10.1016/S1474-4422(12)70191-6, 23079557 PMC3507991

[ref41] Thompson A. E. Voyer D. (2014). Sex differences in the ability to recognise non-verbal displays of emotion: a meta-analysis. Cogn Emot. 28, 1164–1195. doi: 10.1080/02699931.2013.875889, 24400860

[ref42] Venkatesh V. Morris M. Davis G. Davis F. (2003). User acceptance of information technology: toward a unified view. MIS Q. 27, 425–478. doi: 10.2307/30036540

[ref43] Völter C. Peter Thomas J. Maetzler W. Guthoff R. Grunwald M. Hummel T. (2021). Sensory dysfunction in old age. Dtsch. Arztebl. Int. 118, 512–520. doi: 10.3238/arztebl.m2021.0212 34158149 PMC8476826

[ref44] Wada S. Honma M. Masaoka Y. Yoshida M. Koiwa N. Sugiyama H. . (2021). Volume of the right supramarginal gyrus is associated with a maintenance of emotion recognition ability. PLoS One 16:e0254623. doi: 10.1371/JOURNAL.PONE.0254623, 34293003 PMC8297759

[ref45] Wagner M. Watson D. G. (2010). Experimental and theoretical advances in prosody: a review. Lang. Cogn. Process. 25, 905–905. doi: 10.1080/01690961003589492, 22096264 PMC3216045

[ref46] Wang L. Lou Y. Jiang Q. Wang H. Huang S. Xie Y. . (2025). Association between multimorbidity trajectories and subsequent cognitive decline: evidence from two nationwide longitudinal cohorts. J. Affect. Disord. 391:119958. doi: 10.1016/j.jad.2025.119958, 40683537

[ref47] Wang X. T. Wang Z. T. Hu H. Y. Qu Y. Wang M. Shen X. N. . (2021). Association of subjective cognitive decline with risk of cognitive impairment and dementia: a systematic review and meta-analysis of prospective longitudinal studies. J. Prev Alzheimers Dis. 8, 277–285. doi: 10.14283/jpad.2021.27, 34101784 PMC12280825

[ref48] Wei M. Y. Levine D. A. Zahodne L. B. Kabeto M. U. Langa K. M. (2020). Multimorbidity and cognitive decline over 14 years in older Americans. J Gerontol Ser A. 75, 1206–1213. doi: 10.1093/gerona/glz147, 31173065 PMC7243582

[ref49] Yıldırım C. Düzenli-Öztürk S. Parlak M. M. (2024). Assessing the perception of emotional prosody in healthy ageing. Int. J. Lang. Commun. Disord. 59, 2497–2515. doi: 10.1111/1460-6984.13097, 39137279

[ref50] Zhou Y. You Y. Zhang Y. Zhang Y. Yuan C. Xu X. (2025). Multimorbidity and risk of dementia: a systematic review and meta-analysis of longitudinal cohort studies. J. Prev Alzheimers Dis. 12:100164. doi: 10.1016/j.tjpad.2025.100164, 40246681 PMC12321622

[ref51] Zysberg L. (2012). Loneliness and emotional intelligence. J. Psychol. 146, 37–46. doi: 10.1080/00223980.2011.574746 22303611

